# Comparative effectiveness of early initiation of oral nonsteroidal anti-inflammatory drug and oral acetaminophen therapies on the time to knee replacement in patients with knee osteoarthritis in Japan

**DOI:** 10.1186/s12891-023-06415-9

**Published:** 2023-04-14

**Authors:** Shingo Higa, Ken Nakata, Yusuke Karasawa, Kazuhiro Ohwaki

**Affiliations:** 1 Teikyo University Graduate School of Public Health, Tokyo, Japan; 2 Medical Affairs, Viatris Pharmaceuticals Japan Inc., Tokyo, Japan; 3 Department of Health and Sport Sciences, Osaka University Graduate School of Medicine, Osaka, Japan

**Keywords:** Knee osteoarthritis, Nonsteroidal anti-inflammatory drugs, Acetaminophen, Knee replacement, Claims data, Cohort study

## Abstract

**Background:**

Although disease-modifying properties of nonsteroidal anti-inflammatory drugs (NSAIDs) for osteoarthritis (OA) have been reported, the effects of NSAIDs on OA progression remain controversial. The purpose of this study was to investigate the effect of early initiation of oral NSAID therapy on the progression of knee OA.

**Methods:**

In this retrospective cohort study, we extracted data of patients newly diagnosed with knee OA between November 2007 and October 2018 from a Japanese claims database. The primary outcome was the time to knee replacement (KR), and the secondary outcome was the time to composite event including joint lavage and debridement, osteotomy, or arthrodesis in addition to KR. Weighted Cox regression analysis with standardized mortality/morbidity ratio (SMR) weight was performed to compare the outcomes between patients prescribed oral NSAID (NSAID group) and those prescribed oral acetaminophen (APAP) (APAP group) early after a diagnosis of knee OA. Propensity scores were calculated using logistic regression conditioned on potential confounding factors, and SMR weights were calculated using the propensity scores.

**Results:**

The study population comprised 14,261 patients, who were divided into two groups as follows: 13,994 in the NSAID group and 267 in the APAP group. The mean ages of patients in the NSAID and APAP groups were 56.9 and 56.1 years, respectively. Furthermore, 62.01% and 68.16% patients in the NSAID and APAP groups, respectively, were female. The NSAID group had a reduced risk of KR compared with the APAP group in the analysis using SMR weighting (SMR-weighted hazard ratio, 0.19; 95% confidence interval, 0.05–0.78). While no statistically significant difference was found for the risk of composite event between the two groups (SMR-weighted hazard ratio, 0.56; 95% confidence interval, 0.16–1.91).

**Conclusions:**

The risk of KR in the NSAID group was significantly lower than that in the APAP group after accounting for residual confounding using SMR weighting. This finding suggests that oral NSAID therapy early after the initial diagnosis is associated with a reduced risk of KR in patients with symptomatic knee OA.

**Supplementary Information:**

The online version contains supplementary material available at 10.1186/s12891-023-06415-9.

## Background

Osteoarthritis (OA) is a degenerative joint disease that affects the cartilage and its surrounding tissues, and it is characterized by loss of articular cartilage, subchondral bone sclerosis, osteophyte formation, and synovitis [1]. Knee OA is one of the most prevalent joint diseases and a major cause of disability among older people [2, 3]. Because knee OA is accompanied by gradually worsening symptoms, such as chronic pain, stiffness, and restricted range of motion [4], it limits patients’ activities of daily living [5]. Knee OA is also associated with an economic burden by increasing the total health expenditure, of which the cost of joint replacement surgery accounts for a substantial percentage [6, 7].

According to a meta-analysis of 73 studies regarding knee OA prevalence, an estimate of approximately 654.1 million individuals aged ≥ 40 years had knee OA in 2020 globally [8]. In Japan, a large cohort study reported that approximately 25.3 million and 7.8 million people have radiographic and symptomatic knee OA, respectively, and showed that the prevalence of knee OA increased with age [9]. Considering the individual and socioeconomic burden of knee OA in addition to an aging society in developed countries, including Japan, establishing more effective treatment methods that can reduce the burden of knee OA is necessary.

Pharmacological therapies for OA, such as acetaminophen (*N*-acetyl-*p*-aminophenol [APAP]), nonsteroidal anti-inflammatory drugs (NSAIDs), opioids, serotonin–noradrenaline reuptake inhibitors, intra-articular injections of corticosteroids, and intra-articular injections of hyaluronic acid, are effective in pain relief, but these are considered symptomatic treatments. Despite active research and development of disease-modifying OA drugs to slow or reverse OA progression, no disease-modifying OA drugs have been currently approved by regulatory authorities or applied in clinical settings [10].

NSAIDs have been reported to suppress the pathogenesis of cartilage degeneration and destruction induced by synovitis and affect bone remodeling in vitro and in vivo, suggesting the disease-modifying properties of NSAIDs that are mainly used as symptom-modifying drugs [11, 12]. However, the effects of NSAIDs on OA progression remain controversial. Several open-label interventional studies have reported that 12 months of oral treatment with NSAIDs did not affect knee cartilage loss compared with the historical control or knee and hip OA progression [13, 14]. In another open-label randomized study, a short-term oral NSAID therapy improved the disease-specific quality of life in parallel with a decrease in pro-inflammatory cytokine levels in the synovial fluid of patients with knee OA [15]. This result suggests the potential suppressive effect of NSAIDs on disease progression because synovitis is reportedly associated with the development of radiographic knee OA in a case–control study [16], and knee joint effusion was shown to be a predictor of subsequent knee replacement (KR) in a longitudinal study [17]. Furthermore, a cohort study that compared the economic and clinical burden between patients with OA who started oral celecoxib, one of NSAIDs, within 6 months after (early initiator) and > 6 months after (late initiator) the diagnosis showed that the healthcare resource use and medical cost were lower in early initiators than in late initiators [18]. In addition, the concept of the short-term use of NSAIDs during flares and the use of a simple analgesic in the long term has been proposed with the aim to achieve an optimal benefit–risk balance and cost-effectiveness according to the evidence of comparative studies between NSAIDs and APAP [19]. From these findings, we hypothesized that oral NSAID therapy to patients with symptomatic knee OA early after the diagnosis may have a positive effect on the knee OA prognosis by suppressing inflammation, which is involved in disease progression. However, few studies have assessed the effect of early initiation of oral NSAID therapy on the progression of knee OA.

In this study, we addressed the aforementioned hypothesis through an observational study using a claims database. Accordingly, we compared the time to surgical interventions between patients who received oral NSAID therapy early after the diagnosis of knee OA and those with oral APAP therapy, which is an analgesic without anti-inflammatory effects often used relatively early after the diagnosis of knee OA [19].

## Methods

### Study design

This retrospective cohort study was conducted using Japanese Health Insurance claims data to investigate the effect of early initiation of oral NSAID therapy on the progression of knee OA by comparing the time to surgical interventions between patients with oral NSAID therapy and those with oral APAP therapy early after a diagnosis of knee OA. Data of patients diagnosed with knee OA from November 2007 to October 2018 were extracted from a Japanese employment-based health insurance database covering individuals working in companies across Japan and their dependents.

Oral APAP therapy was used as a comparator of oral NSAID therapy because APAP is a widely used analgesic for the treatment of mild or moderate knee OA and often used relatively early after the diagnosis in Japan [20]. In addition, APAP, without an anti-inflammatory effect, is considered appropriate as a comparator in assessing the disease-modifying properties of oral NSAID therapy, which is partially derived from its anti-inflammatory action. Surgical interventions for knee OA were used as a proxy of disease progression because they reflect persistent pain and functional impairment caused by knee OA, and they have been used as outcomes in previous studies using medical claims data [21–23].

### Data sources

This study used data extracted from a Japanese employment-based health insurance database managed by MinaCare Co., Ltd. (Tokyo, Japan). The database contained data for approximately 6.1 million individuals with medical/pharmaceutical claims and approximately 2.4 million individuals with health checkups as of July 2019. The database covers a wide age range up to 74 years, including those working in large-scale companies with branches throughout Japan such as retailing, manufacturing, food, information, transportation, and energy industries, and their dependent family members. Individuals aged ≥ 75 years were not included in this database because they belonged to another medical care system for older senior citizens in Japan.

Medical and pharmaceutical claims data recorded between November 1, 2007, and October 31, 2018, were extracted and used for this study.

### Study population

Patients with a diagnosis of knee OA were identified, and the date of the initial diagnosis of knee OA for each patient was defined as the index date. Moreover, the month of the initial knee OA diagnosis was defined as the index month. Exposure was determined by the presence of a prescription of study drugs (oral NSAIDs or APAP) in the period from the index month to 5 months after the index month. The first day of the month 6 months after the index month was defined as the observation start date (t_0_). According to a previous study in which an early initiator was defined as individuals who received a prescription within 6 months after the diagnosis [18], the threshold for early initiation of study drugs was set at 6 months, which corresponded to 5 months after the index month.

The study population only included patients with knee OA aged ≥ 40 years with at least a 6-month pre-index and 1-month post-t_0_ insurance coverage period. The requirement of having at least a 6-month pre-index insurance coverage period was set to restrict the study population to those who were supposed to be newly diagnosed with knee OA. The age restriction was set based on the low prevalence of knee OA and few indications for surgical intervention in patients aged < 40 years [9].

Patients were included if they were prescribed either of the study drug (oral NSAIDs or APAP) as systemic analgesic during the period from the index month to 5 months after the index month and had ≥ 30 days of exposure period of the study drug. A threshold of ≥ 30 days of exposure period was set considering the administration period of analgesics for knee OA in clinical trials and clinical settings. Conversely, patients who were prescribed both oral NSAID and oral APAP therapies, or other systemic analgesics with an approved indication for OA, suppository or injection of NSAIDs, suppository or injection of APAP, oral form of an extract from inflammatory rabbit skin inoculated by vaccinia virus (an analgesic derived from the non-protein fraction extracted from the inflamed skin of rabbits after vaccinia virus administration, which has been approved and used for pain for a long time in Japan), duloxetine, strong opioids, or weak opioids during abovementioned period were not included in the study population. These criteria were set to limit the study population to patients who were supposed to have symptomatic and mild-to-moderate knee OA to increase the sample homogeneity.

Patients were excluded if they were diagnosed with severe chronic kidney disease, liver failure, other diseases indicated for KR, such as post-traumatic arthritis, knee fracture, rheumatoid arthritis, osteonecrosis, neoplasm of the lower limbs, or Paget’s disease of the bone before t_0_. Patients who had undergone surgical interventions, namely joint lavage/debridement, osteotomy, unicompartmental knee arthroplasty (UKA), total knee arthroplasty (TKA), or arthrodesis, which were defined as outcomes in this study, before t_0_ were also excluded. Detailed definitions of drugs, diagnoses, and medical procedures related to the inclusion and exclusion criteria are described in Supplementary Tables 1–3, respectively.

### Exposure

**Fig. 1:**
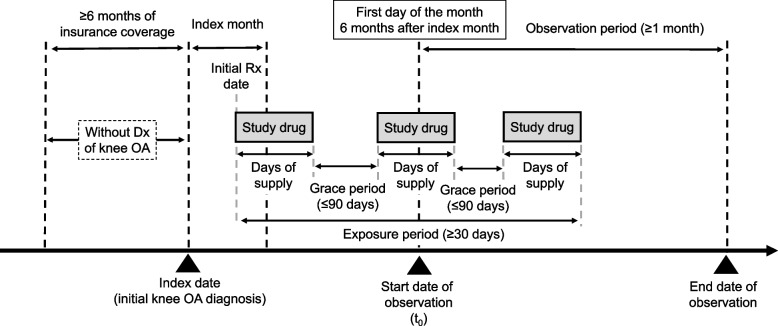
Overview of the study design. Dx, diagnosis; Rx, drug prescription

Exposure was determined by the presence of prescription of either study drug (orally administered NSAIDs or APAP) during the period from the index month to 5 months after the index month (Supplementary Table 1). If a patient was prescribed oral NSAIDs during the above period and the exposure period was ≥ 30 days, the patient was included in the NSAID group. Similarly, if a patient was prescribed oral APAP during the above period and the exposure period was ≥ 30 days, the patient was included in the APAP group. The exposure period was calculated by counting the number of days from the date of initial prescription after the index date to the end date of the last continuous prescription (Fig. 1). The absence of a succeeding prescription by the end of the supply plus a 90-day grace period was considered cessation of treatment at that point.

### Outcomes

Surgical interventions for knee OA were used as outcomes because they have been commonly used as outcome measures in previous studies using similar data sources [21–23]. The primary outcome measure was the time from t_0_ to KR, namely, UKA or TKA. The secondary outcome measure was the time from t_0_ to a composite event (CE), which included medical procedures of joint lavage and debridement, osteotomy, UKA, TKA, or arthrodesis. Detailed definitions of medical procedures related to outcome measures are described in Supplementary Table 3. Patients were followed up from t_0_ to the date of the first outcome event, last date of data extraction, or date of the last claim, whichever came first for each outcome measure.

### Analysis

In the present study, propensity-score weighting using standardized mortality/morbidity ratio (SMR) weight was used to control confounding [24]. Analysis using the SMR weight provides estimates of the change in the average risk of the exposed subgroup produced by the exposure, which corresponds to the exposed group as the standard population [24]. The relative risk estimates of surgical interventions in the NSAID group in comparison with the APAP group, when the NSAID group was used as the standard population, were obtained by using SMR weighting.

The propensity score, which predicts the probability of receiving oral NSAID therapy for each patient, was calculated using logistic regression conditioned on the potential confounding factors, as described below. The following covariates at baseline, including risk factors of knee OA progression and/or knee OA-related pain and factors influencing the likelihood of undergoing KR and pharmacological/nonpharmacological therapies considered effective for knee OA, were considered potential confounding factors based on previous studies [21, 22]: (1) demographic factors: age at the index date, sex, and year of the first diagnosis of knee OA (index year); (2) prescriptions for medications with an approved indication for knee OA treatment (from 6 months to 1 month before the index month): oral, topical, suppository, and injection forms of NSAIDs and oral, suppository, and injection forms of APAP; topical form of other anti-inflammatory analgesics; oral form of an extract from inflammatory rabbit skin inoculated by vaccinia virus; duloxetine; oral, topical, and injection forms of strong opioids; oral and injection forms of weak opioids; and injections of hyaluronic acid, corticosteroid, and chondroitin; (3) diagnosis (prior to 1 month before the index month): obesity, crystal-induced arthritis, cerebrovascular disease, cancer, arrhythmia, deep vein thrombosis, hypertension, ischemic heart disease, valvular disease, hyperlipidemia, diabetes mellitus, heart failure, osteoporosis, hydrarthrosis, sepsis, infectious arthropathies, osteomyelitis, platelet dysfunction/thrombocytopenia, depression, anxiety disorder, and migraine; (4) medical procedures (from 6 months to 1 month before the index month): musculoskeletal rehabilitation and orthotic treatment. Detailed definitions of drugs, diagnoses, and medical procedures related to the covariates are described in Supplementary Tables 1–3, respectively.

The baseline characteristics of the NSAID and APAP groups in the original population and those in the pseudopopulation created by SMR weighting were compared using standardized mean differences (SMDs) [25, 26]. An absolute value of SMD ≥ 0.1 was regarded as an imbalance. The distributions of drugs with approved indications for knee OA used during the observation period in the NSAID and APAP groups were also described. The number of events, person-years of the observation period, and event rate were summarized for each exposure and outcome measure.

Unadjusted and adjusted relative risks of each outcome measure (time to KR and time to CE) in the NSAID group in comparison with those of the APAP group were calculated. The Cox proportional hazard model was used to estimate the crude hazard ratio (HR) and corresponding 95% confidence interval (CI). The weighted Cox proportional hazards model using SMR weighting with a robust variance estimator was used to estimate the SMR-weighted HR and corresponding 95% CI (main analysis). The covariates adjusted by SMR weighting are listed in Supplementary Table 4. The robustness of the study findings was assessed through sensitivity analyses, which used a 60- or 120-day grace period to calculate the exposure period or which used the inverse probability of treatment (IPT) weight [27] instead of the SMR weight.

An exploratory analysis using the propensity-score matching method instead of propensity-score weighting was also conducted to consider the possibility of residual confounding in the main analysis. A one-to-many propensity-score matching (ratio of the NSAID group to the APAP group = 10:1) was used in this analysis to increase precision and control confounding because of a large difference in the number of patients between the NSAID and APAP groups [28]. Another exploratory analysis was conducted using the weighted Cox proportional hazards model with covariates as explanatory variables in which imbalance remained between the two groups in the pseudopopulation.

All statistical analyses were performed using SAS Version 9.4 (SAS Institute, Cary, NC). A *P* value of < 0.05 was considered statistically significant.

#### Ethics statement

This study used data in an anonymized structured format and did not contain personal information. Therefore, informed consent and ethical approval were not required because studies exclusively using unlinkable anonymized data are outside the scope of the ethical guidelines for medical and health research involving human subjects set by the Japanese government. In addition, this study was conducted in accordance with legal and regulatory requirements, e.g., privacy protection laws, and scientific purpose, value, and rigor.

## Results

### Study population and characteristics

**Fig. 2:**
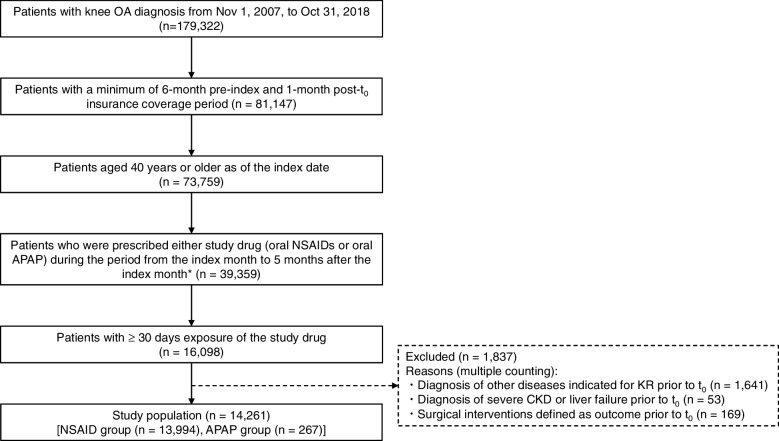
Flowchart of the selection of the study population. * Patients who were prescribed both oral NSAID and oral APAP therapies or other systemic analgesics with an approved indication for OA were not included. APAP, *N*-acetyl-*p*-aminophenol (acetaminophen); CKD, chronic kidney disease; KR, knee replacement; NSAIDs, nonsteroidal anti-inflammatory drugs

A total of 73,759 patients with knee OA aged ≥ 40 years with at least a 6-month pre-index and 1-month post-t_0_ insurance coverage period were included in the present study. Among these patients, 59,400 were prescribed one or more analgesics other than topical NSAIDs/anti-inflammatory analgesics. Of them, 39,359 (66.3%) were prescribed only oral NSAIDs or oral APAP (as systemic analgesics). After the exclusion criteria, 14,261 patients with at least 30 days of exposure to the study drug comprised the study population. The median [1st quartile (Q1) and 3rd quartile (Q3)] of the pre-index insurance coverage period in the study population was 24 (13, 40) months. Of the 14,261 patients, 13,994 and 267 were included in the NSAID and APAP groups, respectively (Fig. 2). The mean age of patients in the NSAID and APAP groups was 56.9 and 56.1 years, respectively. Furthermore, 62.01% and 68.16% patients in the NSAID and APAP groups, respectively, were female (Table 1).

**Table 1:** Baseline characteristics of the NSAID and APAP groups

Characteristics	NSAID group (*N* = 13,994)	APAP group (*N* = 267)	SMD
Age (years), mean (SD)	56.91 (8.85)	56.10 (9.27)	0.089
40–49 years old	3284 (23.47)	73 (27.34)	− 0.089
50–59 years old	5110 (36.52)	91 (34.08)	0.051
60–69 years old	4189 (29.93)	79 (29.59)	0.008
70–74 years old	1411 (10.08)	24 (8.99)	0.037
Female	8678 (62.01)	182 (68.16)	− 0.129
Index year 2009	8 (0.06)	1 (0.37)	− 0.068
Index year 2010	514 (3.67)	5 (1.87)	0.110
Index year 2011	1842 (13.16)	24 (8.99)	0.133
Index year 2012	2375 (16.97)	35 (13.11)	0.108
Index year 2013	2004 (14.32)	32 (11.99)	0.069
Index year 2014	2262 (16.16)	43 (16.10)	0.002
Index year 2015	2109 (15.07)	37 (13.86)	0.034
Index year 2016	1855 (13.26)	52 (19.48)	− 0.169
Index year 2017	1011 (7.22)	38 (14.23)	− 0.228
Index year 2018	14 (0.10)	0 (0)	0.045
NSAIDs (oral)	6003 (42.90)	67 (25.09)	0.383
APAP (oral)	1232 (8.80)	93 (34.83)	− 0.664
NSAIDs (suppository)	210 (1.50)	1 (0.37)	0.117
NSAIDs (injection)	72 (0.51)	1 (0.37)	0.021
APAP (suppository)	12 (0.09)	1 (0.37)	− 0.060
APAP (injection)	25 (0.18)	2 (0.75)	− 0.084
Extract from inflammatory rabbit skin inoculated by vaccinia virus (oral)	119 (0.85)	4 (1.50)	− 0.060
Duloxetine	25 (0.18)	0 (0)	0.060
Strong opioid (oral)	0 (0)	0 (0)	−
Strong opioid (injection)	297 (2.12)	9 (3.37)	− 0.076
Strong opioid (topical)	3 (0.02)	0 (0)	0.021
Weak opioid (oral)	293 (2.09)	7 (2.62)	− 0.035
Weak opioid (injection)	3 (0.02)	0 (0)	0.021
Hyaluronic acid (injection)	188 (1.34)	5 (1.87)	− 0.042
Chondroitin (injection)	52 (0.37)	0 (0)	0.086
Corticosteroid (injection)	1360 (9.72)	26 (9.74)	− 0.001
NSAIDs (topical)	4236 (30.27)	74 (27.72)	0.056
Other anti-inflammatory analgesics (topical)	124 (0.89)	5 (1.87)	− 0.085
Obesity	290 (2.07)	9 (3.37)	− 0.080
Crystal-induced arthritis	824 (5.89)	15 (5.62)	0.012
Cerebrovascular disease	1788 (12.78)	39 (14.61)	− 0.053
Cancer	1639 (11.71)	32 (11.99)	− 0.008
Arrhythmia	1580 (11.29)	41 (15.36)	− 0.120
Deep vein thrombosis	175 (1.25)	4 (1.50)	− 0.021
Hypertension	6904 (49.34)	136 (50.94)	− 0.032
Ischemic heart disease	1822 (13.02)	44 (16.48)	− 0.098
Valvular disease	579 (4.14)	13 (4.87)	− 0.035
Hyperlipidemia	6819 (48.73)	143 (53.56)	− 0.097
Diabetes mellitus	4412 (31.53)	86 (32.21)	− 0.015
Heart failure	1048 (7.49)	39 (14.61)	− 0.229
Osteoporosis	1394 (9.96)	33 (12.36)	− 0.076
Hydrarthrosis	70 (0.50)	4 (1.50)	− 0.100
Sepsis	46 (0.33)	2 (0.75)	− 0.057
Infectious arthropathies	19 (0.14)	2 (0.75)	− 0.093
Osteomyelitis	23 (0.16)	1 (0.37)	− 0.041
Platelet dysfunction/thrombocytopenia	195 (1.39)	7 (2.62)	− 0.088
Depression	1627 (11.63)	33 (12.36)	− 0.023
Anxiety disorder	1490 (10.65)	36 (13.48)	− 0.087
Migraine	812 (5.80)	24 (8.99)	− 0.122
Musculoskeletal rehabilitation	1231 (8.80)	32 (11.99)	− 0.105
Orthotic treatment	189 (1.35)	3 (1.12)	0.021

All data are shown as n (%) unless specified

*APAP N*-acetyl-*p*-aminophenol (acetaminophen), *NSAID* nonsteroidal anti-inflammatory drug, *SD* standard deviation, *SMD* standardized mean difference

Several differences were noted in the baseline characteristics between the two groups, and 14 covariates had an absolute SMD value ≥ 0.1. Specifically, at baseline, the NSAID group was more likely to be prescribed with oral NSAIDs (42.90% vs. 25.09%, SMD = 0.383) but less likely with oral APAP (8.80% vs. 34.83%, SMD =  − 0.664) than the APAP group (Table 1). The medians (Q1, Q3) of the exposure period, which indicates continuous prescription of the study drug, in the NSAID and APAP groups were 87 (49, 164) and 74 (49, 115) days, respectively. The baseline characteristics of the NSAID and APAP groups after SMR weighing (pseudopopulation) are shown in Table 2. Although slight imbalances (0.1 ≤ absolute SMD value < 0.2) between the NSAID and APAP groups remained in nine covariates, the baseline characteristics of both groups in the pseudopopulation were more comparable than those in the original population.

**Table 2:** Characteristics of the NSAID and APAP groups after standardized mortality ratio weighting (Pseudopopulation)

Characteristics	NSAID group (*N* = 13,994)	APAP group (*N* = 14,704.5)	SMD
Age (years), mean, (SD)	56.91 (8.85)	56.30 (66.67)	0.013
40–49 years old	3284 (23.47)	3253.7 (22.13)	0.032
50–59 years old	5110 (36.52)	6264.1 (42.60)	− 0.125
60–69 years old	4189 (29.93)	3563.0 (24.23)	0.129
70–74 years old	1411 (10.08)	1623.8 (11.04)	− 0.031
Female	8678 (62.01)	8565.4 (58.25)	0.077
Index year 2009	8 (0.06)	6.9 (0.05)	0.005
Index year 2010	514 (3.67)	599.8 (4.08)	− 0.021
Index year 2011	1842 (13.16)	1506.4 (10.25)	0.091
Index year 2012	2375 (16.97)	3093.9 (21.04)	− 0.104
Index year 2013	2004 (14.32)	2050.3 (13.94)	0.011
Index year 2014	2262 (16.16)	2563.5 (17.43)	− 0.034
Index year 2015	2109 (15.07)	2333.4 (15.87)	− 0.022
Index year 2016	1855 (13.26)	1597.4 (10.86)	0.074
Index year 2017	1011 (7.22)	952.9 (6.48)	0.029
Index year 2018	14 (0.1)	0 (0)	0.045
NSAIDs (oral)	6003 (42.90)	6800.2 (46.25)	− 0.067
APAP (oral)	1232 (8.80)	1078.9 (7.34)	0.054
NSAIDs (suppository)	210 (1.50)	17.4 (0.12)	0.155
NSAIDs (injection)	72 (0.51)	190.6 (1.30)	− 0.083
APAP (suppository)	12 (0.09)	8.0 (0.05)	0.012
APAP (injection)	25 (0.18)	7.7 (0.05)	0.037
Extract from inflammatory rabbit skin inoculated by vaccinia virus (oral)	119 (0.85)	46.7 (0.32)	0.070
Duloxetine	25 (0.18)	0 (0)	0.060
Strong opioid (oral)	0 (0)	0 (0)	−
Strong opioid (injection)	297 (2.12)	393.5 (2.68)	− 0.036
Strong opioid (topical)	3 (0.02)	0 (0)	0.021
Weak opioid (oral)	293 (2.09)	60.0 (0.41)	0.152
Weak opioid (injection)	3 (0.02)	0 (0)	0.021
Hyaluronic acid (injection)	188 (1.34)	70.8 (0.48)	0.091
Chondroitin (injection)	52 (0.37)	0 (0)	0.086
Corticosteroid (injection)	1360 (9.72)	1002.9 (6.82)	0.105
NSAIDs (topical)	4236 (30.27)	3798.3 (25.83)	0.099
Other anti-inflammatory analgesics (topical)	124 (0.89)	34.5 (0.24)	0.087
Obesity	290 (2.07)	164.1 (1.12)	0.076
Crystal-induced arthritis	824 (5.89)	904.3 (6.15)	− 0.011
Cerebrovascular disease	1788 (12.78)	1393.5 (9.48)	0.105
Cancer	1639 (11.71)	1673.4 (11.38)	0.010
Arrhythmia	1580 (11.29)	1629.9 (11.09)	0.007
Deep vein thrombosis	175 (1.25)	127.2 (0.87)	0.038
Hypertension	6904 (49.34)	6637.1 (45.14)	0.084
Ischemic heart disease	1822 (13.02)	1982.2 (13.48)	− 0.014
Valvular disease	579 (4.14)	724.7 (4.93)	− 0.038
Hyperlipidemia	6819 (48.73)	7238.4 (49.23)	− 0.010
Diabetes mellitus	4412 (31.53)	3881.5 (26.40)	0.113
Heart failure	1048 (7.49)	1226.7 (8.34)	− 0.032
Osteoporosis	1394 (9.96)	1304.5 (8.87)	0.037
Hydrarthrosis	70 (0.50)	39.0 (0.27)	0.038
Sepsis	46 (0.33)	34.6 (0.24)	0.018
Infectious arthropathies	19 (0.14)	11.9 (0.08)	0.017
Osteomyelitis	23 (0.16)	31.4 (0.21)	− 0.011
Platelet dysfunction/thrombocytopenia	195 (1.39)	116.0 (0.79)	0.058
Depression	1627 (11.63)	1849.0 (12.57)	− 0.029
Anxiety disorder	1490 (10.65)	1568.6 (10.67)	− 0.001
Migraine	812 (5.80)	979.3 (6.66)	− 0.035
Musculoskeletal rehabilitation	1231 (8.80)	1129.7 (7.68)	0.041
Orthotic treatment	189 (1.35)	35.5 (0.24)	0.125

All data are shown as n (%) unless specified

*APAP N*-acetyl-*p*-aminophenol (acetaminophen), *NSAID* nonsteroidal anti-inflammatory drug, *SD* standard deviation, *SMD* standardized mean difference

Except for the study drugs (oral NSAIDs and APAP), the proportions of patients who were prescribed each drug with an approved indication for knee OA treatment during the observation period were comparable between the two groups (Supplementary Table 5).

### Outcome data and main results

The number of events, total person-years during the observation period, and event rates for each exposure and outcome measure are summarized in Table 3. The medians (Q1, Q3) of the observation period for the primary outcome measure were 18 (8, 35) and 16 (7, 31) months in the NSAID and APAP groups, respectively. The crude incidence rates of KR in the NSAID and APAP groups were 1.70 and 6.30 per 1000 person-years, respectively. The crude incidence rates of CE in the NSAID and APAP groups were 5.76 and 12.66 per 1000 person-years, respectively.

**Table 3:** Incidence of surgical interventions in the NSAID and APAP group

Group	Total (N)	KR (n)	PY for KR	KR rate /1000 PY	CE (n)	PY for CE	CE rate /1000 PY
NSAID	13,994	46	27,105.8	1.70	155	26,919.0	5.76
APAP	267	3	476.0	6.30	6	473.8	12.66

Knee replacement included unicompartmental knee arthroplasty and total knee arthroplasty. Composite event included surgical interventions of joint lavage, joint debridement, osteotomy, unicompartmental knee arthroplasty, total knee arthroplasty, and arthrodesis

*APAP N*-Acetyl-*p*-aminophenol (acetaminophen), *CE* composite event, *KR* knee replacement, *NSAID* nonsteroidal anti-inflammatory drug, *PY* person-years

The NSAID group had a reduced risk of KR compared with the APAP group in the analysis using SMR weighting (SMR-weighted HR 0.19; 95% CI 0.05–0.78). However, no significant difference was found in the risk of CE between the two groups (SMR-weighted HR 0.56; 95% CI 0.16–1.91) (Table 4).

**Table 4:** Comparison of estimated risk of surgical interventions between the NSAID and APAP groups

Group	Endpoint	Unadjusted, HR (95% CI)	*P* value	SMR weighted, HR (95% CI)	*P* value
NSAID vs APAP	Knee replacement	0.26 (0.08–0.82)	0.022	0.19 (0.05–0.78)	0.021
	Composite event	0.46 (0.20–1.03)	0.060	0.56 (0.16–1.91)	0.352

Knee replacement included unicompartmental knee arthroplasty and total knee arthroplasty. Composite event included surgical interventions of joint lavage, joint debridement, osteotomy, unicompartmental knee arthroplasty, total knee arthroplasty, and arthrodesis

*APAP N*-acetyl-*p*-aminophenol (acetaminophen), *CI* confidence interval, *HR* hazard ratio, *NSAID* nonsteroidal anti-inflammatory drug, *SMR* standardized mortality/morbidity ratio

### Sensitivity and exploratory analysis

The sensitivity analyses used a 60- or 120-day grace period instead of a 90-day grace period for the calculation of the exposure period, and the sensitivity analysis using IPT weighting showed that the NSAID group was associated with a reduced risk of KR compared with the APAP group. In addition, no significant difference was found in the risk of CE between the two groups. These results were similar to those of the main analysis (Supplementary Table 6).

A small imbalance in some covariates remained between the pseudopopulation of the NSAID and APAP groups after SMR weighting. The covariates were age categories, index year, suppository NSAIDs, oral weak opioids, corticosteroid injection, cerebrovascular disease, diabetes mellitus, and orthotic treatment. Post-hoc exploratory analyses using a propensity-score matching and a weighted Cox proportional hazard model with these covariates as explanatory variables produced estimates similar to those of the main analysis (Supplementary Table 7).

## Discussion

This study demonstrated that the prescription of oral NSAIDs early after diagnosis was associated with approximately 80% reduction in KR risk compared with the prescription of oral APAP in patients with symptomatic knee OA. As KR is a hard outcome of knee OA that predicts OA progression [29], this finding suggests that oral NSAID therapy early after the initial diagnosis is associated with a reduced risk of KR in patients with symptomatic knee OA.

Conversely, no significant difference was found in the risk of CE, comprising joint lavage and debridement, osteotomy, UKA, TKA, and arthrodesis, between the NSAID group and APAP group, although the point estimate of SMR-weighted HR was < 1. CE was set as the secondary outcome because it was considered a more sensitive surrogate marker of OA progression than KR alone [21]. However, joint lavage/debridement, one of the events included in the CE, is often performed for short-term symptom relief in the early stage of knee OA [30]. Therefore, this surgery may reflect mainly clinical symptoms rather than OA progression and contributed to a decrease in the difference between the NSAID and APAP groups, if the assumption that early initiation of oral NSAID therapy has beneficial effects on the progression of knee OA is right. This would be a possible reason for the inconsistency between the results of the primary and secondary outcomes.

Although some studies have evaluated the effects of NSAIDs on the progression of knee OA, the disease-modifying properties of NSAIDs remain controversial. A cohort study using data from the Osteoarthritis Initiative (OAI) reported that long-term NSAID use was associated with slowing of joint space narrowing in patients with knee OA, although the association was not statistically significant [31]. Conversely, other following studies reported the deleterious effects of analgesics including NSAIDs in the natural course of knee OA. A cohort study using data from OAI suggested that long-term use of analgesics is associated with radiographic progression of knee OA and increased risk of KR [32]. Another observational study using data from OAI reported that current users of NSAIDs were associated with a loss of medial minimum joint space width compared with non-current users [33]. However, these studies have not provided insights regarding the effect of minimal or short-term use of NSAIDs on the progression of knee OA. In this study, since the exposure period of NSAIDs was relatively short (median, 87 days), its findings may be interpreted as the effects of short-term use of NSAIDs on the progression of knee OA unlike those in previous studies, which have proposed a new perspective that short-term oral NSAID therapy at earlier timing may be beneficial to the prognosis of knee OA.

Results of a previous cohort study may support the findings of the present study. The study used a claims database compared the economic and clinical burden between patients with OA who started celecoxib within 6 months after the diagnosis (early initiators) and those who started > 6 months after diagnosis (late initiators) and showed that healthcare resource use and all-cause and OA-related medical costs were significantly lower in early initiators [18]. The OA-related cost reduction may be partly explained by the reduced risk of KR brought by early initiation of NSAIDs, because inpatient claims accounted for approximately 80% of OA-related costs in the study and KR is a very expensive medical procedure.

The disease-modifying effects of NSAIDs on cartilage, synovium, and bone are reportedly mediated by the regulation of prostaglandins, cytokines, matrix metalloproteinase, and tissues [12]. Synovitis is characterized by the production of inflammatory cytokines, such as interleukin (IL)-1, IL-6, tumor necrosis factor (TNF)-α, and vascular endothelial growth factor (VEGF), infiltration of mononuclear cells, thickening of the synovial lining layer, and fibrosis that appears from the early stage of OA, and is associated with symptoms and structural progression including cartilage damage in OA [34]. As a short-term oral NSAID therapy significantly decreased the concentrations of IL-6, TNF-α, and VEGF in the synovial fluid [15], the anti-inflammatory effects of NSAIDs are likely one of the mechanisms of its beneficial effects on the progression of knee OA. Subchondral bone remodeling also plays an important role in OA pathogenesis and progression [35, 36]. The early stages of OA are characterized by increased vascularity and reduced bone density. By contrast, late-stage OA is characterized by decreased bone resorption without a decrease in bone formation and development of subchondral sclerosis [36]. An in vivo animal study demonstrated that bisphosphonates protected articular cartilage deterioration and prevented osteophyte formation, which suggests that drugs that inhibit bone remodeling have potential disease-modifying properties in OA [35]. An in vitro study showed that NSAIDs have suppressive actions on both osteoblasts and osteoclasts, that is, they may inhibit bone resorption and formation [12]. Therefore, NSAIDs may be useful in the early-stage OA when subchondral bone remodeling is enhanced and may delay OA progression. The effect of NSAIDs on subchondral bone remodeling is another possible explanation of the beneficial effect of NSAIDs on the progression of knee OA. Moreover, the superior pain-improving effect of NSAIDs to APAP could contribute to the longer time to KR in the NSAID group than in the APAP group [37], because pain intensity may affect the time to KR.

Knee OA is a slowly progressing disorder. Accordingly, long-term follow-up is necessary to grasp the progression to KR in most patients with knee OA. Since the median observation periods for the primary outcome measure in this study were 18 and 16 months in the NSAID and APAP groups, respectively, we could evaluate only relatively short-term effects of NSAIDs on the progression of knee OA. However, some proportions of patients with knee OA experience rapid progression from pre-radiographic disease to advanced-stage radiographic disease and are highly probable to undergo KR within a short period [38, 39]. The early use of anti-inflammatory therapies and physical rehabilitation focused on neuromuscular control may be beneficial for people with these types of knee OA because joint effusion and synovitis are key contributors that accelerate joint decline by a vicious cycle [39]. Considering the presence of patients with rapidly progressing knee OA, the difference in the risk of undergoing a KR between the NSAID and APAP groups in this study may mean that the anti-inflammatory effect of NSAIDs breaks the vicious cycle caused by effusion and synovitis and slows down the progression of knee OA in patients at risk of rapid progression.

The latest 2019 American College of Rheumatology guideline strongly recommends topical and oral NSAID therapy and positions oral NSAID therapy as the initial oral treatment of choice for OA. However, it conditionally recommends APAP for those with limited pharmacologic options because of intolerance of or contraindications to NSAIDs to control knee OA symptoms [40]. Similarly, the current 2019 Osteoarthritis Research Society International guidelines strongly recommend topical NSAID therapy because of the minimal and mild adverse events and conditionally recommend oral NSAID therapy as the treatment option, followed by topical NSAID therapy. On the contrary, APAP is not recommended due to the lack of supportive evidence [41]. The American Academy of Orthopaedic Surgeons guideline published in 2021 recommends oral NSAID and APAP therapies. However, it comments that providers may consider using oral treatment with NSAIDs instead of acetaminophen when there were no contraindications to oral NSAID therapy because it provided a more significant reduction in pain and improved function than acetaminophen [42]. In contrast to the above guidelines, the National Institute for Health and Care Excellence guidance recommends APAP as the first choice of pharmacological therapy and stated that APAP and/or topical treatment with NSAIDs should be considered ahead of oral NSAID therapy considering the side effects of NSAIDs. However, the updated version of this guidance describes that the ongoing evidence review identified reduced effectiveness of APAP in the management of OA compared with what was previously thought [43]. Thus, although recommendations for oral NSAID and APAP therapies for OA are not the same among guidelines, oral NSAID therapy is regarded as more effective than oral APAP therapy for the management of OA symptoms.

The findings of this study suggest that early initiation of oral NSAID therapy instead of oral APAP therapy may be more beneficial if topically administered NSAIDs are insufficient for pain relief and/or function improvement in patients with symptomatic knee OA, considering the potential disease-modifying properties of NSAIDs. This approach aligns with the recommendations of pharmacological management stated in the above OA guidelines, although the recommendations were created based on symptom management, not on disease modification of OA.

### Limitations

This study has several limitations. First, because the data source is a medical and pharmaceutical claims database, some major risk factors for the onset and/or progression of knee OA such as body mass index, physical activities, knee extensor muscle strength, and knee alignment [44–51] were unavailable. However, to minimize the residual confounding caused by these unmeasured confounding factors, some proxy variables were used in this analysis (e.g., diagnosis of obesity as a proxy of body mass index and musculoskeletal rehabilitation as a proxy of physical activities or knee extensor muscle strength). The variables related to the severity of knee OA, such as pain intensity, functional status, and image findings, were also not available from the claims database. Therefore, the study population was restricted to patients who were assumed to be newly diagnosed and had symptomatic and mild-to-moderate knee OA based on the ingenuity of inclusion criteria to balance the severity of knee OA between comparison groups as much as possible. Baseline analgesics use was adjusted through propensity-score weighting. This study may have not completely excluded patients with a non-new onset of knee OA including those who revisited for pain exacerbation after a certain period without visits. However, the majority of the study population were assumed to be patients with new onset knee OA because of the relatively long pre-diagnosis insurance coverage period (median was 24 months). Although a small imbalance remained between comparison groups after SMR weighting when considering SMD ≥ 0.1 as the cutoff point of imbalance [25], the post-hoc exploratory analysis using propensity-score matching and the weighted Cox proportional hazard model with imbalanced covariates as explanatory variables produced similar estimates to the main analysis. These results suggest that the small imbalance observed in the analysis using SMR weighting does not cause major residual confounding in the main results.

Second, because the database comprised employees and their dependents, it does not cover retired people. People aged ≥ 75 years are not also included in this database because they are enrolled in the public medical insurance system for older senior citizens. Considering this limitation, it may be difficult to generalize the results of this study to all older patients with knee OA. However, a diagnosis of symptomatic knee OA occurs relatively early in life. OA incidence peaks in the group aged 55–64 years, and the median age was 55 years [52]. Therefore, our findings could be applied to a large proportion of patients with knee OA.

Third, to avoid the over-adjustment bias [53] caused by controlling intermediate variables, concomitant drugs for OA treatment during the observation period, were not adjusted in this study. These variables might influence the study outcomes because some drugs have protective effects or deleterious effects on the articular cartilage [54, 55]. However, confounding by these concomitant drugs would not be a major issue because the length of the observation periods and distributions of drugs for OA treatment, except for the study drugs during the observation period, were not substantially different between the comparison groups (Supplementary Table 5).

Fourth, as accurate information on mortality were difficult to extract from the Japanese claims database, a competing risk analysis with death as a competing risk could not be performed. However, the impact of death as a competing risk might not be particularly large, because this data source did not include patients aged ≥ 75 years.

Fifth, the follow-up period of this study was short. Thus, the results of this study might be difficult to generalize, particularly in terms of knee OA subtypes that slowly progresses.

Finally, the sample size in this study, especially the number of patients with knee OA classified into the APAP group, was relatively small to investigate the effectiveness of drugs considering the heterogeneous nature of knee OA. A post-hoc power calculation revealed that the statistical power to detect the difference in the risk of KR between the comparison groups was not very high (39.1%).

## Conclusions

The risk of KR in the NSAID group was significantly lower than that in the APAP group after accounting for residual confounding through SMR weighting. This finding suggests that oral NSAID therapy early after the initial diagnosis is associated with a reduced risk of KR in patients with symptomatic knee OA. Further longer studies using a larger data source that can measure the major potential confounders are required to address the limitations of this study.

## Supplementary Information

**Additional file 1: Supplementary Table 1.** Operational definition of variables related to drugs. **Supplementary Table 2.** Operational definition of variables related to diagnoses. **Supplementary Table 3.** Operational definition of variables related to medical procedures. **Supplementary Table 4.** List of covariates that were adjusted or matched by propensity score method. **Supplementary Table 5.** Distribution of drugs with indication for knee osteoarthritis during the observation period in the NSAID and APAP groups. **Supplementary Table 6.** Sensitivity analysis to compare the estimated risk of surgical interventions between the NSAID and APAP groups. **Supplementary Table 7.** Comparison of estimated risk of surgical interventions between the NSAID and APAP groups (exploratory analysis).

## Data Availability

MinaCare data are proprietary to MinaCare Co., Ltd. and not publicly available for research purposes. Researchers who would like to access the data for research purposes should contact Dr. Yuji Yamamoto (mc_info@minacare.co.jp) to make a data use agreement and pay a data availability fee. The datasets used and/or analyzed during the current study are available from the corresponding author on reasonable request after concluding the data use agreement between the requester and MinaCare Co., Ltd.

## Abbreviations

APAP: *N*-Acetyl-*p*-aminophenol (acetaminophen)
CE: Composite event
CI: Confidence interval
HR: Hazard ratio
KR: Knee replacement
NSAIDs: Nonsteroidal anti-inflammatory drugs
OA: Osteoarthritis
PY: Person-years
SD: Standard deviation
SMD: Standardized mean difference
SMR: Standardized mortality/morbidity ratio
TKA: Total knee arthroplasty
UKA: Unicompartmental knee arthroplasty
