# Supplementary material for: Comparative effectiveness of early initiation of oral nonsteroidal anti-inflammatory drug and oral acetaminophen therapies on the time to knee replacement in patients with knee osteoarthritis in Japan
Source: BMC Musculoskelet Disord. 2023 Apr 14;24:297. doi: 10.1186/s12891-023-06415-9 (PMC10103473; doi:10.1186/s12891-023-06415-9)
Supplement: Supplementary file 1 — Additional file 1: Supplementary Table 1. Operational definition of variables related to drugs. Supplementary Table 2. Operational definition of variables related to diagnoses. Supplementary Table 3. Operational definition of variables related to medical procedures. Supplementary Table 4. List of covariates that were adjusted or matched by propensity score method. Supplementary Table 5. Distribution of drugs with indication for knee osteoarthritis during the observation period in the NSAID and APAP groups. Supplementary Table 6. Sensitivity analysis to compare the estimated risk of surgical interventions between the NSAID and APAP groups. Supplementary Table 7. Comparison of estimated risk of surgical interventions between the NSAID and APAP groups (exploratory analysis). [file 12891_2023_6415_MOESM1_ESM.docx]

Supplementary Table 1. Operational definition of variables related to drugs

| Variable | Generic drug name | Drug code ^a^ |
| --- | --- | --- |
| NSAIDs (oral) | Celecoxib | 1149037 |
|  | Aspirin | 1143001 |
|  | Mefenamic acid | 1141005 |
|  | Flufenamate aluminum | 1141004 |
|  | Diclofenac sodium | 1147002 |
|  | Amfenac sodium | 1147006 |
|  | Indomethacin | 1145001 |
|  |  | 1145002 |
|  | Acemetacin | 1145003 |
|  | Indomethacin farnesil | 1145005 |
|  | Proglumetacin maleate | 1145004 |
|  | Sulindac | 1149015 |
|  | Etodolac | 1149032 |
|  | Nabumetone | 1149027 |
|  | Ibuprofen | 1149001 |
|  | Flurbiprofen | 1149011 |
|  | Naproxen | 1149007 |
|  | Pranoprofen | 1149010 |
|  | Tiaprofenic acid | 1149025 |
|  | Oxaprozin | 1149026 |
|  | Loxoprofen sodium hydrate | 1149019 |
|  | Zaltoprofen | 1149029 |
|  | Piroxicam | 1149017 |
|  | Ampiroxicam | 1149030 |
|  | Lornoxicam | 1149036 |
|  | Meloxicam | 1149035 |
|  | Tiaramide hydrochloride | 1148001 |
|  | Epirizole | 1148003 |
|  | Emorphazone | 1148004 |
|  | Tenoxicam | 1149021 |
|  | Bucolome | 1149009 |
| APAP (oral) | Acetaminophen | 1141001 |
|  |  | 1141007 |
| APAP (suppository) | Acetaminophen | 1141700 |
| APAP (injection) | Acetaminophen | 1141400 |
| NSAIDs (suppository) | Diclofenac sodium | 1147700 |
|  | Indomethacin | 1145700 |
|  | Ketoprofen | 1149700 |
|  | Piroxicam | 1149701 |
| NSAIDs (injection) | Flurbiprofen axetil | 1149407 |
|  | Ketoprofen | 1149402 |
| NSAIDs (topical) | Indomethacin | 2649719 |
|  | Piroxicam | 2649730 |
|  | Felbinac | 2649731 |
|  | Loxoprofen sodium hydrate | 2649735 |
|  | Diclofenac sodium | 2649734 |
|  | Ketoprofen | 2649729 |
|  | Esflurbiprofen | 2649896 |
|  | Flurbiprofen | 2649732 |
| Other anti-inflammatory analgesics  (topical) | Heparinoid / adrenal extract / salicylic acid | 2649865 |
|  | Methyl salicylate / dl−camphor / l−menthol | 2649895 |
|  | Methyl salicylate / dl−camphor /capsicum extract | 2649843 |
|  | Methyl salicylate / l-menthol / dl-camphor / glycyrrhetinic acid | 2649858 |
|  | Methyl salicylate / glycol salicylate / diphenhydramine | 2649851 |
|  | Methyl salicylate / diphenhydramine | 2649859 |
| Extract from inflammatory rabbit skin inoculated by vaccinia virus (oral) | Extract from inflammatory rabbit skin inoculated by vaccinia virus | 1149023 |
| Hyaluronic acid (injection) | Purified sodium hyaluronate | 3999408 |
|  | Sodium hyaluronate crosslinked polymer | 3999428 |
| Corticosteroid (injection) | Hydrocortisone sodium succinate | 2452400 |
|  | Methylprednisolone sodium succinate | 2456405 |
|  | Methylprednisolone acetate | 2456402 |
|  | Triamcinolone acetonide | 2454402 |
|  | Dexamethasone sodium phosphate | 2454405 |
|  | Dexamethasone sodium metasulfobenzoate | 2454401 |
|  | Betamethasone sodium phosphate | 2454404 |
|  | Betamethasone acetate / betamethasone sodium phosphate | 2454408 |
| Duloxetine | Duloxetine hydrochloride | 1179052 |
| Strong opioid (oral) | Morphine hydrochloride hydrate | 8114003 |
|  | Morphine hydrochloride hydrate sustained-release preparation | 8114005 |
|  | Morphine sulfate hydrate sustained-release preparation | 8114004 |
|  | Oxycodone hydrochloride hydrate | 8119002 |
|  | Oxycodone hydrochloride hydrate sustained-release preparation | 8119002 |
|  | Fentanyl citrate | 8219001 |
|  | Pethidine hydrochloride | 8211001 |
| Strong opioid (injection) | Morphine hydrochloride hydrate | 8114401 |
|  | Morphine / atropine | 8119505 |
|  | Oxycodone hydrochloride hydrate | 8119400 |
|  | Compound oxycodone | 8119500 |
|  | Compound oxycodone/ atropine | 8119504 |
|  | Fentanyl citrate | 8219400 |
|  | Pethidine hydrochloride | 8211400 |
|  | Buprenorphine hydrochloride | 1149403 |
| Strong opioid (topical) | Fentanyl citrate | 8219701 |
|  | Fentanyl | 8219700 |
|  | Buprenorphine hydrochloride | 1149703 |
|  | Buprenorphine | 1149704 |
| Weak opioid (oral) | Tramadol hydrochloride | 1149038 |
|  | Tramadol hydrochloride / acetaminophen | 1149117 |
|  | Codeine phosphate hydrate | 2242001 |
|  | Dihydrocodeine phosphate | 2242002 |
| Weak opioid (injection) | Tramadol hydrochloride | 1149400 |
| Chondroitin (injection) | Chondroitin Sulfate Sodium | 3991400 |
|  | Chondroitin Sulfate Sodium / Sodium Salicylate | 1149500 |
| ^a^ Seven upper digits of the Japanese National Health Insurance drug price list code.  APAP, *N*-acetyl-*p*-aminophenol (acetaminophen); NSAIDs, nonsteroidal anti-inflammatory drugs. | | |

Supplementary Table 2. Operational definition of variables related to diagnoses

| Variable | Type of code | ICD10 classification name /  disease name | ICD10 code /  disease code |
| --- | --- | --- | --- |
| Knee osteoarthritis | Disease code | Knee osteoarthritis | 7153018 |
| Post-traumatic arthritis of knee | Disease code | Post-traumatic arthritis of knee, bilateral | 8841043 |
|  |  | Post-traumatic arthritis of knee, unilateral | 8830542 |
|  |  | Post-traumatic arthritis of knee | 8831716 |
|  |  | Traumatic arthritis of knee | 8845244 |
| Knee fracture | ICD10 | Fracture of patella | S820 |
|  |  | Fracture of upper end of tibia | S821 |
|  |  | Fracture of lower end of femur | S724 |
| Rheumatoid arthritis | ICD10 | Seropositive rheumatoid arthritis | M05 |
|  |  | Other rheumatoid arthritis | M06 |
| Osteonecrosis | ICD10 | Osteonecrosis | M87 |
| Neoplasm of long bones of lower limb | ICD10 | Malignant neoplasm of long bones of lower limb | C402 |
|  | ICD10 | Benign neoplasm of long bones of lower limb | D162 |
| Paget disease of bone | ICD10 | Paget disease of bone | M88 |
| Severe chronic kidney disease | Disease code | Chronic kidney disease, stage 4 | N184 |
|  |  | Chronic kidney disease, stage 5 | N185 |
| Hepatic failure | ICD10 | Hepatic failure, not elsewhere classified | K72 |
| Obesity | ICD10 | Obesity | E66 |
| Crystal-induced arthritis | ICD10 | Gout | M10 |
|  |  | Other crystal arthropathies | M11 |
| Cerebrovascular diseases | ICD10 | Cerebrovascular diseases | I60-I69 |
| Cancer | ICD10 | Malignant neoplasms | C00-C97 |
| Arrhythmia | ICD10 | Atrioventricular and left bundle-branch block | I44 |
|  |  | Other conduction disorders | I45 |
|  |  | Cardiac arrest | I46 |
|  |  | Paroxysmal tachycardia | I47 |
|  |  | Atrial fibrillation and flutter | I48 |
|  |  | Other cardiac arrhythmias | I49 |
| Deep vein thrombosis | ICD10 | Phlebitis and thrombophlebitis | I80 |
|  |  | Portal vein thrombosis | I81 |
|  |  | Other venous embolism and thrombosis | I82 |
| Hypertension | ICD10 | Essential (primary) hypertension | I10 |
|  |  | Secondary hypertension | I15 |
| Ischemic heart diseases | ICD10 | Ischemic heart diseases | I20-I25 |
| Valvular disease | ICD10 | Rheumatic mitral valve diseases | I05 |
|  |  | Rheumatic aortic valve diseases | I06 |
|  |  | Rheumatic tricuspid valve diseases | I07 |
|  |  | Multiple valve diseases | I08 |
|  |  | Nonrheumatic mitral valve disorders | I34 |
|  |  | Nonrheumatic aortic valve disorders | I35 |
|  |  | Nonrheumatic tricuspid valve disorders | I36 |
|  |  | Pulmonary valve disorders | I37 |
|  |  | Endocarditis, valve unspecified | I38 |
|  |  | Endocarditis and heart valve disorders in diseases classified elsewhere | I39 |
| Hyperlipidemia | ICD10 | Disorders of lipoprotein metabolism and other lipidemias | E78 |
| Diabetes mellitus | ICD10 | Diabetes mellitus | E10-E14 |
| Heart failure | ICD10 | Heart failure | I50 |
| Osteoporosis | ICD10 | Osteoporosis with pathological fracture | M80 |
|  |  | Osteoporosis without pathological fracture | M81 |
|  |  | Osteoporosis in diseases classified elsewhere | M82 |
| Hydrarthrosis | Disease code | Intermittent hydrarthrosis | 8831502 |
|  |  | Hydrarthrosis | 8831600 |
|  |  | Knee hydrarthrosis | 7190013 |
| Sepsis | ICD10 | Streptococcal sepsis | A40 |
|  |  | Other sepsis | A41 |
| Infectious arthropathies | ICD10 | Pyogenic arthritis | M00 |
|  |  | Direct infections of joint in infectious and parasitic diseases classified elsewhere | M01 |
|  |  | Reactive arthropathies | M02 |
|  |  | Postinfective and reactive arthropathies in diseases classified elsewhere | M03 |
| Osteomyelitis | ICD10 | Osteomyelitis | M86 |
| Platelet dysfunction / thrombocytopenia | ICD10 | Purpura and other hemorrhagic conditions | D69 |
| Depression | ICD10 | Depressive episode | F32 |
|  |  | Recurrent depressive disorder | F33 |
| Anxiety disorder | ICD10 | Phobic anxiety disorders | F40 |
|  |  | Other anxiety disorders | F41 |
| Migraine | ICD10 | Migraine | G43 |
| ICD10 according to ICD-10 version 2010.  Disease code according to the Health Insurance Claims Review & Reimbursement Services of Japan. | | | |

Supplementary Table 3. Operational definition of variables related to medical procedures

| Variable | Medical procedure | Medical procedure code |
| --- | --- | --- |
| Arthrodesis | Invasive arthrodesis (knee) | 150047210 |
| Joint lavage / debridement | Synovectomy (knee) | 150037310 |
|  | Arthroscopic synovectomy (knee) | 150310410 |
|  | Arthrectomy (knee) | 150041910 |
|  | Removal of loose bodies (knee) | 150039310 |
|  | Debridement (knee) | 150048410 |
|  | Arthroscopic removal of loose bodies (knee) | 150312410 |
|  | Arthroscopic removal of foreign bodies (including inserts) in joint (knee) | 150309510 |
|  | Removal of foreign bodies (including inserts) in joint (knee) | 150036410 |
|  | Arthroscopic meniscectomy | 150313110 |
|  | Arthroscopic meniscal repair | 150313210 |
|  | Meniscectomy | 150040910 |
|  | Meniscal repair | 150261910 |
| Osteotomy | Osteotomy (lower leg bone) | 150027910 |
|  | Osteotomy (femur) | 150027710 |
| Knee replacement including TKA and UKA (outcome) | Artificial joint replacement (knee) | 150050510 |
| Knee replacement including TKA and UKA (exclusion criteria) | Artificial joint replacement (knee) | 150050510 |
|  | Revision artificial joint replacement (knee) | 150256110 |
| Musculoskeletal rehabilitation | Musculoskeletal rehabilitation fee | 180032710 |
|  |  | 180027810 |
|  |  | 180027910 |
|  |  | 180034510 |
|  |  | 180034610 |
|  |  | 180034710 |
|  |  | 180044030 |
|  |  | 180044130 |
|  |  | 180044230 |
|  |  | 180045810 |
|  |  | 180045910 |
|  |  | 180046010 |
|  |  | 180052730 |
|  |  | 180052830 |
|  |  | 180052930 |
|  |  | 180053030 |
|  |  | 180053130 |
|  |  | 180053230 |
|  |  | 180053330 |
|  |  | 180053430 |
|  |  | 180053530 |
|  |  | 180053630 |
|  |  | 180053730 |
|  |  | 180053830 |
| Orthotic treatment | Therapeutic orthosis casting method | 140047950 |
|  | Prostheses casting method | 140047850 |
|  | Cast for therapeutic orthosis casting | 140047210 |
|  |  | 140047310 |
| TKA, total knee arthroplasty; UKA, unicompartmental knee arthroplasty. | | |

Supplementary Table 4. List of covariates that were adjusted or matched by propensity score method

| Category | Covariates |
| --- | --- |
| Demographic factor | Age at the index date |
|  | Sex |
|  | Index year |
| Drug  (From 6 months to 1 month before the index month) | NSAIDs (oral) |
|  | NSAIDs (topical) |
|  | NSAIDs (suppository) |
|  | NSAIDs (injection) |
|  | APAP (oral) |
|  | APAP (suppository) |
|  | APAP (injection) |
|  | Other anti-inflammatory analgesics (topical) |
|  | Extract from inflammatory rabbit skin inoculated by vaccinia virus (oral) |
|  | Duloxetine |
|  | Strong opioid (oral) |
|  | Strong opioid (topical) |
|  | Strong opioid (injection) |
|  | Weak opioid (oral) |
|  | Weak opioid (injection) |
|  | Hyaluronic acid (injection) |
|  | Corticosteroid (injection) |
|  | Chondroitin (injection) |
| Diagnosis  (Prior to 1 month before the index month) | Obesity |
|  | Crystal-induced arthritis |
|  | Cerebrovascular disease |
|  | Cancer |
|  | Arrhythmia |
|  | Deep vein thrombosis |
|  | Hypertension |
|  | Ischemic heart disease |
|  | Valvular disease |
|  | Hyperlipidemia |
|  | Diabetes mellitus |
|  | Heart failure |
|  | Osteoporosis |
|  | Hydrarthrosis |
|  | Sepsis |
|  | Infectious arthropathies |
|  | Osteomyelitis |
|  | Platelet dysfunction/thrombocytopenia |
|  | Depression |
|  | Anxiety disorder |
|  | Migraine |
| Medical procedure  (From 6 months to 1 month before the index month) | Musculoskeletal rehabilitation |
|  | Orthotic treatment |

APAP, N-acetyl-p-aminophenol (acetaminophen); NSAIDs, nonsteroidal anti-inflammatory drugs.

Supplementary Table 5. Distribution of drugs with indication for knee osteoarthritis during the observation period in the NSAID and APAP groups

| Class of analgesics | NSAID group  (N = 13,994) | APAP group  (N = 267) |
| --- | --- | --- |
| NSAIDs (oral) | 10546 (75.36) | 144 (53.93) |
| APAP (oral) | 4129 (29.51) | 175 (65.54) |
| NSAIDs (suppository) | 761 (5.44) | 16 (5.99) |
| NSAIDs (injection) | 335 (2.39) | 6 (2.25) |
| APAP (suppository) | 16 (0.11) | 1 (0.37) |
| APAP (injection) | 218 (1.56) | 5 (1.87) |
| An extract from inflammatory rabbit skin inoculated by vaccinia virus (oral) | 469 (3.35) | 8 (3.00) |
| Duloxetine | 176 (1.26) | 3 (1.12) |
| Strong opioid (oral) | 31 (0.22) | 1 (0.37) |
| Strong opioid (injection) | 1274 (9.10) | 28 (10.49) |
| Strong opioid (topical) | 37 (0.26) | 1 (0.37) |
| Weak opioid (oral) | 1215 (8.68) | 30 (11.24) |
| Weak opioid (injection) | 6 (0.04) | 0 (0) |
| Hyaluronic acid (injection) | 3564 (25.47) | 48 (17.98) |
| Chondroitin (injection) | 152 (1.09) | 1 (0.37) |
| Corticosteroid (injection) | 3861 (27.59) | 86 (32.21) |
| NSAIDs (topical) | 8931 (63.82) | 166 (62.17) |
| Other anti-inflammatory analgesics (topical) | 378 (2.70) | 7 (2.62) |
| All data are shown as n (%).  APAP, *N*-acetyl-*p*-aminophenol (acetaminophen); NSAID, nonsteroidal anti-inflammatory drug. | | |

Supplementary Table 6. Sensitivity analysis to compare the estimated risk of surgical interventions between the NSAID and APAP groups

| Change from main analysis | Group | Endpoint | Unadjusted,  HR (95% CI) | *P*  value | Weighted,  HR (95% CI) | *P*  value |
| --- | --- | --- | --- | --- | --- | --- |
| Grace period (60 days) | NSAID (N = 12,544)  vs APAP (N = 194) | KR | 0.20 (0.06-0.64) | 0.007 | 0.18 (0.04-0.71) ^a^ | 0.014 |
|  |  | CE | 0.34 (0.15-0.76) | 0.009 | 0.51 (0.15-1.74) ^a^ | 0.285 |
| Grace period (120 days) | NSAID (N = 15,063)  vs APAP (N = 312) | KR | 0.28 (0.09-0.91) | 0.034 | 0.21 (0.05-0.84) ^a^ | 0.027 |
|  |  | CE | 0.51 (0.23-1.16) | 0.108 | 0.63 (0.19-2.08) ^a^ | 0.444 |
| Analysis using IPTW | NSAID (N = 13,994)  vs APAP (N = 267) | KR | 0.26 (0.08-0.82) | 0.022 | 0.19 (0.05-0.78) ^b^ | 0.021 |
|  |  | CE | 0.46 (0.20-1.03) | 0.060 | 0.55 (0.16-1.88) ^b^ | 0.344 |
| Knee replacement included unicompartmental knee arthroplasty and total knee arthroplasty. Composite event included surgical interventions of joint lavage and debridement, osteotomy, unicompartmental knee arthroplasty, total knee arthroplasty, and arthrodesis.  ^a^ Analysis using standardized mortality/morbidity ratio (SMR) weight; ^b^ Analysis using IPTW.  APAP, *N*-acetyl-*p*-aminophenol (acetaminophen); CE, composite event; CI, confidence interval; HR, hazard ratio; IPTW, inverse probability of treatment weight; KR, knee replacement; NSAID, nonsteroidal anti-inflammatory drug. | | | | | | |

Supplementary Table 7. Comparison of estimated risk of surgical interventions between the NSAID and APAP groups (exploratory analysis)

| Change from main analysis | Group | Endpoint | Adjusted, HR (95% CI) | *P* value |
| --- | --- | --- | --- | --- |
| Analysis using PS matching (10:1) | NSAID (N = 2,385)  vs APAP (N = 266) | KR | 0.13 (0.02-0.80) ^a^ | 0.027 |
|  |  | CE | 0.49 (0.19-1.25) ^a^ | 0.135 |
| Adjusted imbalanced covariates in pseudopopulation | NSAID (N = 13,994)  vs APAP (N = 267) | KR | 0.25 (0.07-0.88) ^b^ | 0.031 |
|  |  | CE | 0.61 (0.19-1.97) ^b^ | 0.407 |
| Knee replacement included unicompartmental knee arthroplasty and total knee arthroplasty. Composite event included any surgical interventions of joint lavage and debridement, osteotomy, unicompartmental knee arthroplasty, total knee arthroplasty, and arthrodesis.  ^a^ Adjusted by PS matching; ^b^ Analysis using standardized mortality/morbidity ratio (SMR) weight, with added age categories, index year (2012), NSAIDs suppository, oral weak opioid, corticosteroid injection, cerebrovascular disease, diabetes mellitus, and orthotic treatment as explanatory variables.  APAP, *N*-acetyl-*p*-aminophenol (acetaminophen); CE, composite event; CI, confidence interval; HR, hazard ratio; KR, knee replacement; NSAID, non-steroidal anti-inflammatory drug; PS, propensity score. | | | | |
